# Expression of Embryonic Stem Cell Markers on the Microvessels of WHO Grade I Meningioma

**DOI:** 10.3389/fsurg.2018.00065

**Published:** 2018-10-26

**Authors:** Ganeshwaran Shivapathasundram, Agadha C. Wickremesekera, Helen D. Brasch, Reginald Marsh, Swee T. Tan, Tinte Itinteang

**Affiliations:** ^1^Gillies McIndoe Research Institute, Wellington, New Zealand; ^2^Department of Neurosurgery, Wellington Regional Hospital, Wellington, New Zealand; ^3^Department of Psychological Medicine, Auckland University, Auckland, New Zealand; ^4^Wellington Regional Plastic, Maxillofacial and Burns Unit, Hutt Hospital, Wellington, New Zealand

**Keywords:** meningioma, OCT4, NANOG, SOX2, KLF, c-MYC, embryonic, stem cells

## Abstract

**Aim:** The presence of cells within meningioma (MG) that express embryonic stem cell (ESC) markers has been previously reported. However, the precise location of these cells has yet to be determined.

**Methods:** 3,3-Diaminobenzidine (DAB) immunohistochemical (IHC) staining was performed on 11 WHO grade I MG tissue samples for the expression of the ESC markers OCT4, NANOG, SOX2, KLF4 and c-MYC. Immunofluorescence (IF) IHC staining was performed to investigate the localization of each of these ESC markers. NanoString and colorimetric *in situ* hybridization (CISH) mRNA expression analyses were performed on six snap-frozen MG tissue samples to confirm transcriptional activation of these proteins, respectively.

**Results:** DAB IHC staining demonstrated expression of OCT4, NANOG, SOX2, KLF4, and c-MYC within all 11 MG tissue samples. IF IHC staining demonstrated the expression of the ESC markers OCT4, NANOG, SOX2, KLF4, and c-MYC on both the endothelial and pericyte layers of the microvessels. NanoString and CISH mRNA analyses confirmed transcription activation of these ESC markers.

**Conclusion:** This novel finding of the expression of all aforementioned ESC markers in WHO grade I MG infers the presence of a putative stem cells population which may give rise to MG.

## Introduction

Meningioma (MG) accounts for 25–30% of primary intracranial and intraspinal tumors (1), and has been traditionally thought to be derived from arachnoid cap cells of the brain and spinal cord (2) based on the correlational histological and ultrastructural studies comparing arachnoid cap cells with MG cells (2).

Tumor stem cells are proposed to be the cellular origin of cancer including glioblastoma (GB) (3) and leukemia (4), are increasingly thought to be the origin of benign entities such as Dupuytren's disease (5), infantile hemangioma (6) and MG (1, 7–9). Cultured MG cells form tumor spheres (8), demonstrate self-renewal, and express embryonic stem cell (ESC) associated markers including SOX2, nestin (1) and KLF4 (10). Identification and characterization of ESCs within MG may lead to the understanding of the pathogenesis of this tumor. This may result in the development of novel treatment especially for the more difficult, aggressive, recurrent, anaplastic or skull base MGs.

Recent reports have documented the presence of cells with stem cell phenotype within MG, although their exact location has not been clearly defined (1, 7, 8, 10, 11). Stem cells are characterized by their expression of certain ESC-associated markers including OCT4, SOX2, NANOG, KLF4 and c-MYC. OCT4, is a transcription factor that, plays a critical role in conjunction with SOX2 and NANOG in embyrogenesis (12). It is a gatekeeper for ESC pluripotency (12) and is important in tumor locoregional recurrence and metastasis (13). NANOG, a homeobox binding protein found in ESCs that plays a role in transcriptional regulation of self-renewal and pluripotency (12). It is believed to coordinate the self-renewal and pluripotency of ESCs as well as contributing to carcinogenesis and metastasis (11, 12). SOX2, a high-mobility SRY-related HMG box transcription factor involved in multiple signal transduction pathways and implicated in pathological cell proliferation, migration, invasion and tumorigenesis (14). SOX2 promotes stem cell maintenance and pluripotency of cancer stem cells (CSCs) (15, 16). KLF4, a transcription factor involved in cell proliferation, differentiation and apoptosis (17). It is one of the most frequently mutated genes in secretory MG but it also contributes to the self-renewal and pluripotency of ESCs (10, 18). The c-MYC oncoprotein is of critical importance in proliferation and growth of normal and neoplastic cells (19). c-MYC is expressed by recurrent high-grade lesions, while low-grade lesions do not express c-MYC (20).

In this study, we investigated the expression of the ESC-associated markers OCT4, SOX2, NANOG, KLF4 and c-MYC in WHO grade I MG using immunohistochemical (IHC) staining, and colorimetric *in situ* hybridization (CISH) and NanoString mRNA analyses.

## Materials and methods

### Patient samples

WHO grade I MG lesions from ten female and one male patients, aged 36-85 (mean, 61.8) years, were obtained from the Gillies McIndoe Research Institute Tissue Bank for this study which was approved by the Central Region Health and Disability Ethics Committee (ref. no. 15/CEN/28/AM01) with written informed patient consent.

### Immunohiostochemical staining

Four micrometer-thick sections of formalin-fixed paraffin-embedded from all 11 patients were subjected to 3,3-diaminobenzidine (DAB) IHC staining for OCT4 (1:30; cat# MRQ-10, Cell Marque, Rocklin, CA, United States), NANOG (1:100; cat# ab80892, Abcam, Cambridge, MA, United States), SOX2 (1:200; cat# PA1-094, Thermo Fisher Scientific, Rockford, IL, United States), KLF4 (1:200; cat# NBP2-24749SS, Novus Biologicals LLC, Littleton, CO, United States) and c-MYC (1:1,000; ca# 9E10, Abcam). Staining with a mouse (ready-to-use; cat# IR750, Dako, Copenhagen, Denmark) and rabbit (ready-to-use; cat# IR600, Dako) primary antibody isotype control combination was performed as an appropriate negative control, as previously described (21).

MG samples from four of the original cohort of 11 patients subjected to DAB IHC staining underwent immunofluorescence (IF) IHC staining using combinations of smooth muscle actin (SMA, ready-to-use; cat# PA0943, Leica) that marks the pericyte layer, with either NANOG, SOX2, or KLF4; or ERG (ready-to-use; cat# EP111, Cell Marque) that highlights the endothelial layer, with either OCT4 or c-MYC, to determine expression within the microvessels, as previously reported (13). Appropriate positive controls included seminoma for OCT4 and NANOG, skin for SOX2, breast carcinoma for KLF4 and prostate adenocarcinoma for c-MYC.

### Colorimetric *in-situ* hybridization

To confirm protein expression demonstrated by DAB IHC staining we performed CISH tissues on 4 μm-thick sections of formalin-fixed paraffin-embedded MG from six of the original cohort of patients subjected to DAB IHC staining, using probes (Advanced Cell Diagnostics, Newark, CA, United States) for OCT4 (cat# 592868), NANOG (cat# 604498), SOX2 (cat# 477658), KLF4 (cat# 457468) and c-MYC (cat# 311768), with dapB (cat# 312038) as an appropriate negative probe, for detection using the ACD kit (cat# 322100, Advanced Cell Diagnostics). Both IHC and CISH staining was performed on the Leica Bond Rx autostainer (Leica). Positive control tissue for both the IHC and CISH staining were seminoma for OCT4 and NANOG, skin for SOX2, breast carcinoma for KLF4 and prostate adenocarcinoma for c-MYC.

### Nanostring mRNA analysis

RNA was extracted from snap-frozen MG samples of the same six patients used for CISH, were subjected to NanoString mRNA analysis (NanoString Technologies, Seattle, WA, United States) for mRNA transcripts, OCT4 (POU5F1, NM_002701.4), NANOG (NM_024865.2), SOX2 (NM_003106.2), KLF4 (NM_004235.4), c-MYC (NM_002467.3) and the house-keeping gene GusB (NM_000181.1), performed by New Zealand Genomics (Dunedin, New Zealand).

### Image capture and analysis

The DAB IHC and CISH stained images were captured on the Olympus BX53 microscope fitted with an Olympus DP21 digital camera and analyzed with the Cellsens 2.0 software (Olympus, Tokyo, Japan). IF IHC stained images were captured on the Olympus FV1200 biological confocal laser-scanning microscope with subsequent 2D deconvolution using cellSens Dimension 1.11 software (Olympus).

### Statistical analysis

Statistical analysis of the NanoString mRNA data was performed using the *t*-test (SPSS v 24).

## Results

### DAB IHC staining

**Figure 1:**
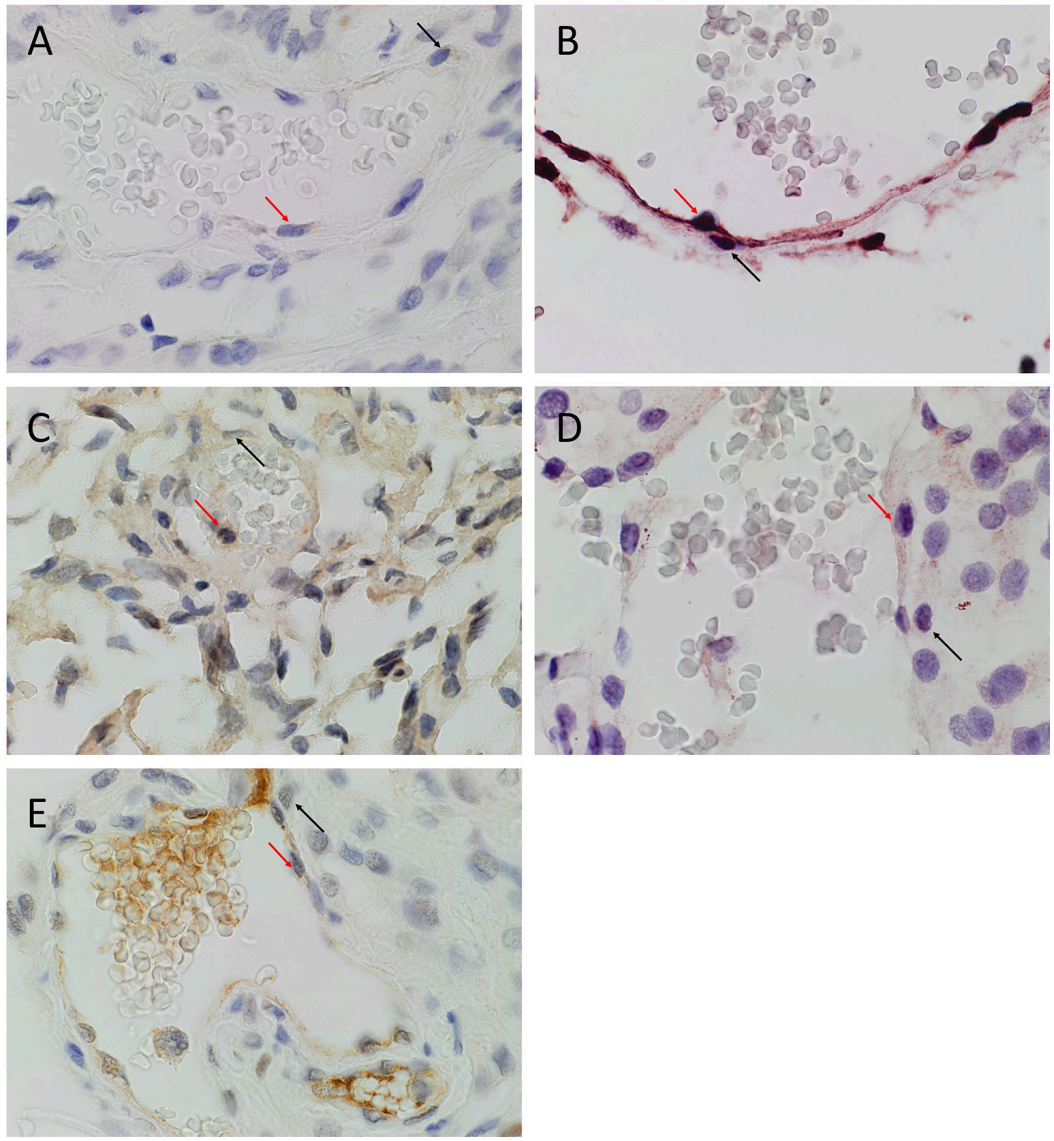
Representative 3,3-diaminobenzidine immunohistochemical stained images of WHO grade I meningioma for OCT4 (**A**, brown), NANOG (**B**, purple), SOX2 (**C**, brown), KLF4 (**D**, purple) and c-MYC (**E**, brown) expressed on the endothelial (*red arrows*) and pericyte (*black arrows*) layers. Cell nuclei were counterstained with hematoxylin (**A-E**, blue). Original magnification: 400X.

DAB IHC staining demonstrated expression of OCT4 (Figure 1A, brown), NANOG (Figure 1B, purple), SOX2 (Figure 1C, brown), KLF4 (Figure 1D, purple) and c-MYC (Figure 1E, brown), most prominently by cells on the endothelial (Figures 1A–E, *red arrows*) and pericyte (Figures 1A–E, *black arrows*) layers of the microvessels within the MG samples. The positive (data not shown) and negative (data not shown) controls demonstrated the expected staining patterns.

### Colorimetric *in-situ* hybridization

**Figure 2:**
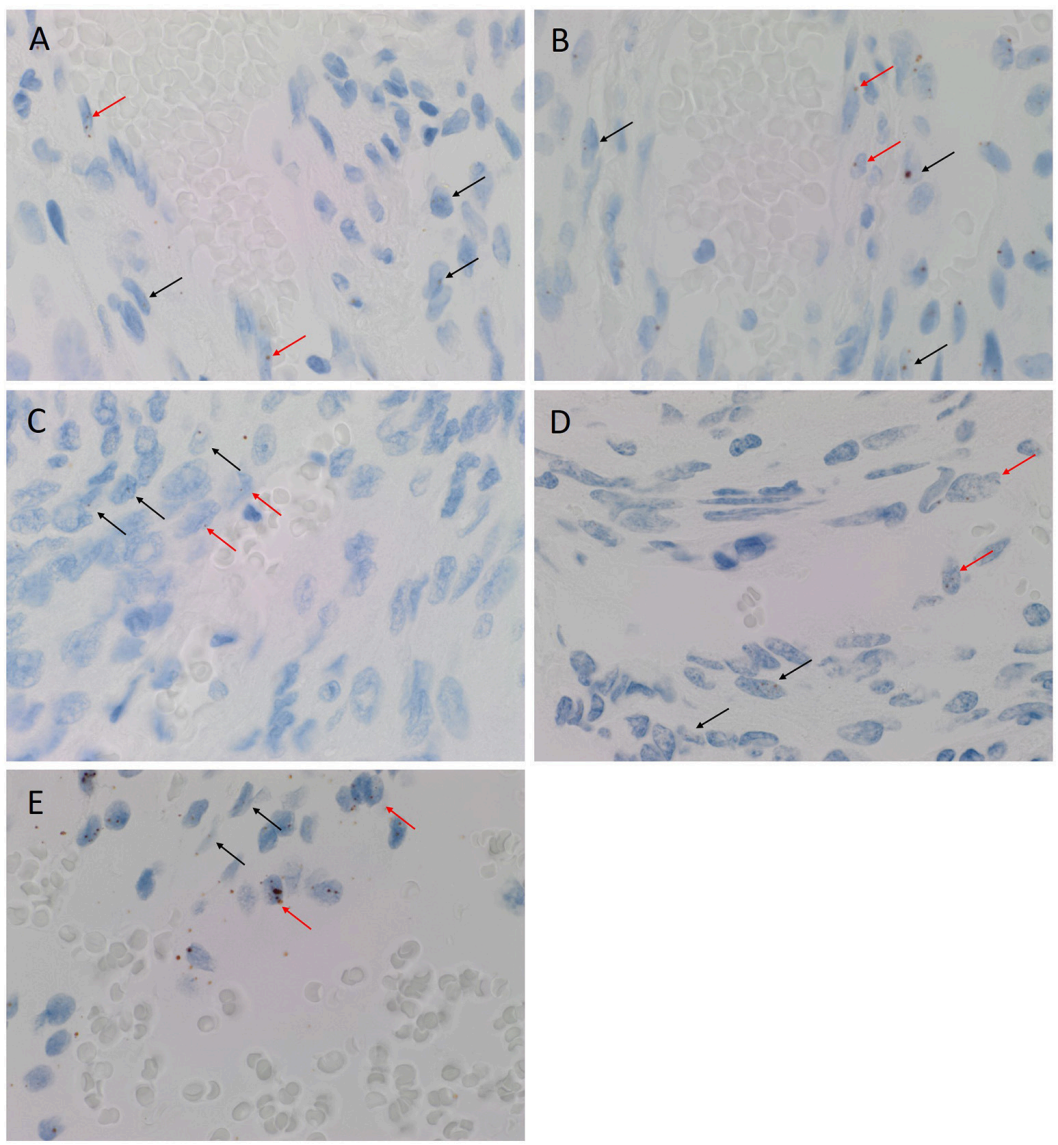
Representative colorimetric *in situ* hybridization stained images of WHO grade I meningioma demonstrating mRNA transcript expression for OCT4 (**A**, brown), NANOG (**B**, brown), SOX2 (**C**, brown), KLF4 (**D**, brown) and c-MYC (**E**, brown). Nuclei were counterstained with hematoxylin (**A-E**, blue). Orignal magnification: 1,000X.

CISH confirmed the expression of OCT4 (Figure 2A, brown), NANOG (Figure 2B, brown), SOX2 (Figure 2C, brown), KLF4 (Figure 2D, brown) and c-MYC (Figure 2E, brown) in both the endothelial (Figures 2A–E, *arrowheads*) and pericyte (Figures 2A–E, *arrows*) layers. Positive (data not shown) and negative (data not shown) controls for CISH showed staining patterns as expected.

### Nanostring mRNA analysis

**Figure 3:**
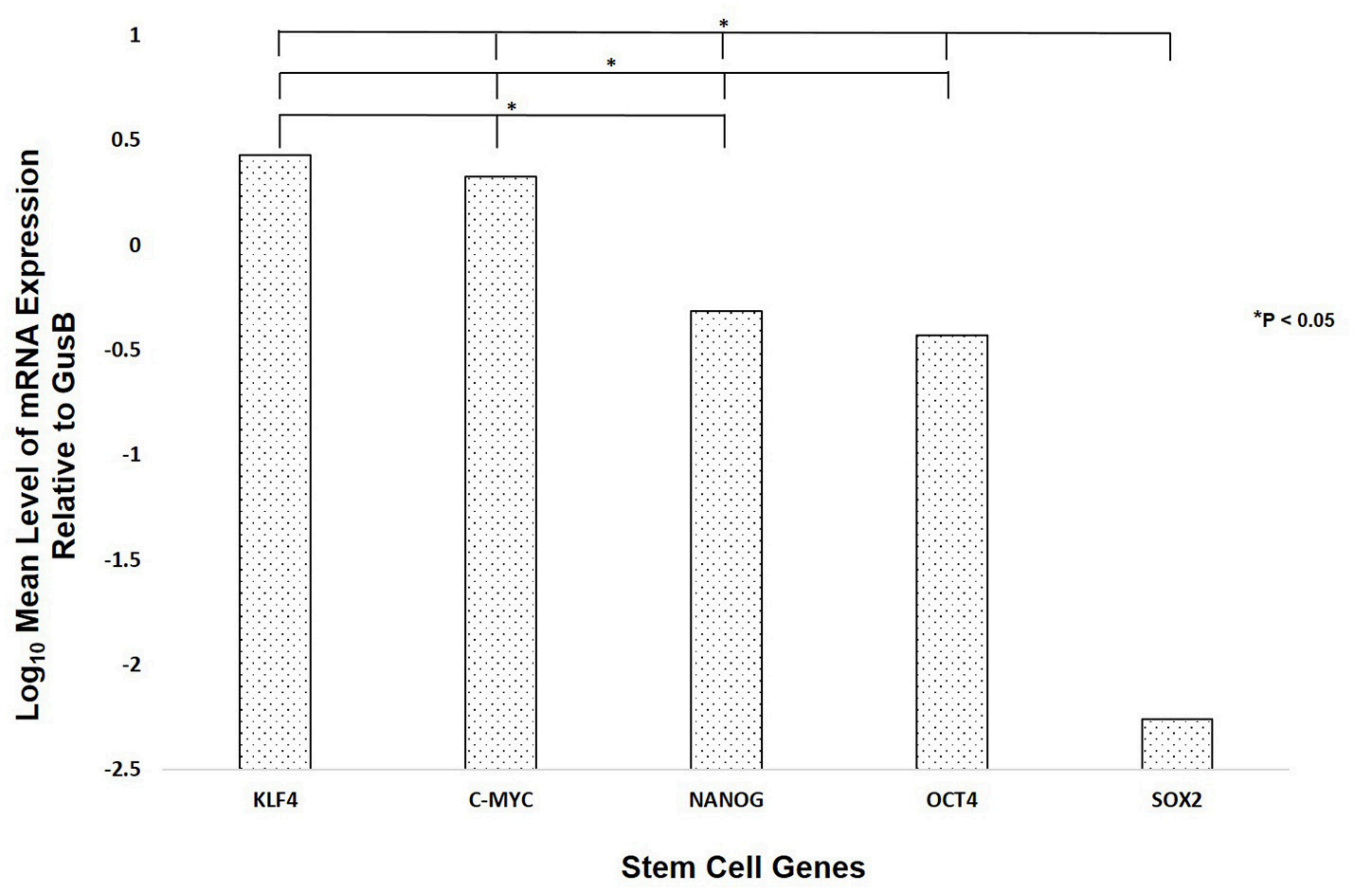
NanoString mRNA analysis of six WHO grade I meningioma samples demonstrating the presence of mRNA transcripts for OCT4, NANOG, SOX2, KLF4, and c-MYC.

NanoString mRNA analysis demonstrated transcriptional activation for all five ESC genes investigated (Figure 3), with significantly high expression of transcripts for KLF4 followed by c-MYC, NANOG, OCT4, and SOX2 (*p* < 0.05).

### IF IHC staining

**Figure 4:**
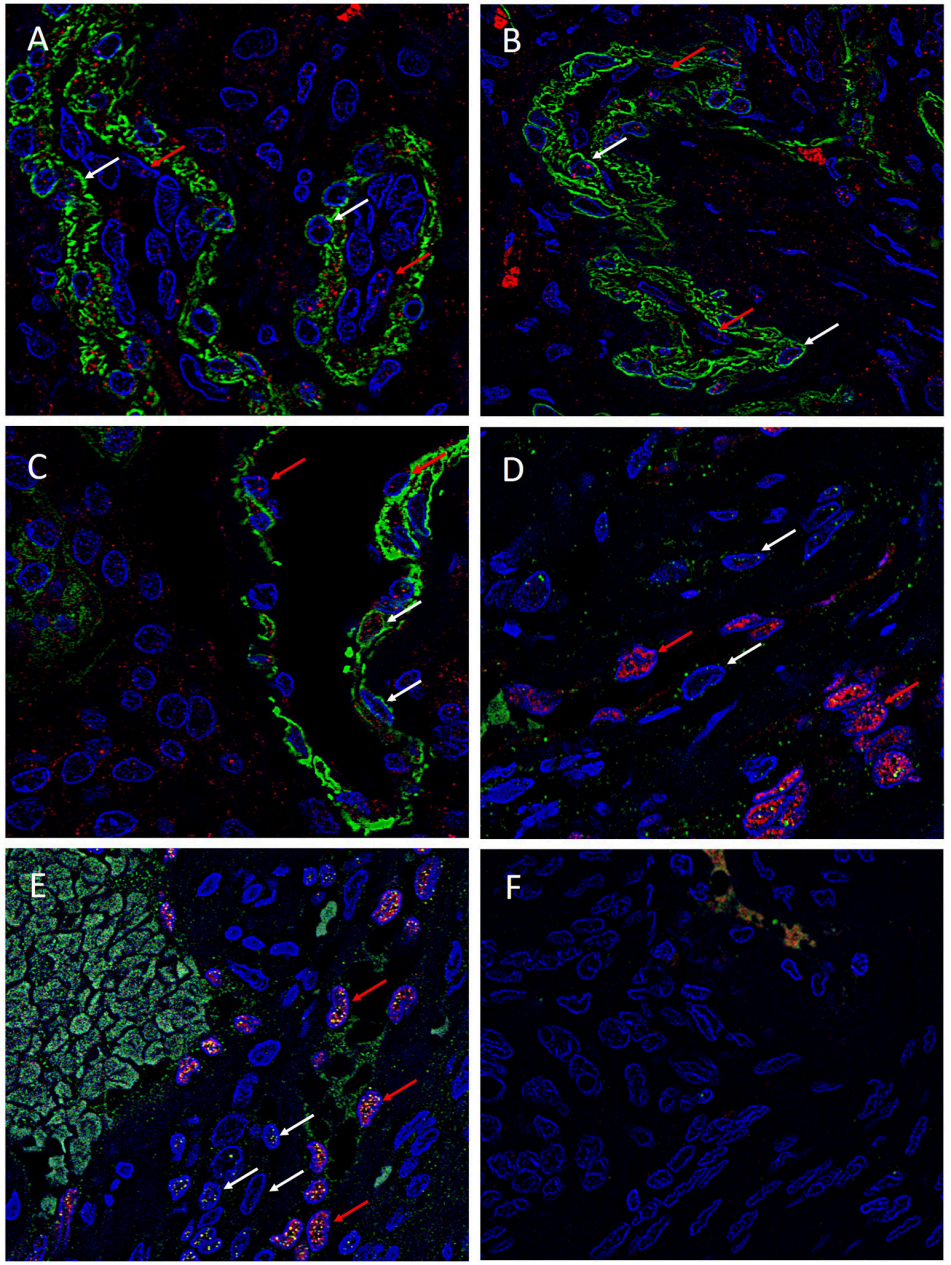
Representative immonoflourescence immunohistochemical stained images of WHO grade I meningioma demonstrating the expression of SOX2 (**A**, red), NANOG (**B**, red), KLF4 (**C**, red), OCT4 (**D**, green) and c-MYC (**E**, green) on both the SMA^+^ (**A–C**, red, *red arrows*) pericyte layer, and the ERG^+^ endothelial layer (**D,E**, red, *white arrows*). Cell nuclei were counterstained with 4′, 6′-diamino-2-phenylindole **(A–F)**, blue. Original magnification: 600X.

To investigate the expression of the ESC markers on either the endothelial or the pericyte layer of the microvessels of the MG lesions, we used smooth muscle actin (SMA) and ERG (22) to differentiate between the pericyte and endothelial layers, respectively. SOX2 (Figure 4A, red), NANOG (Figure 4B, red), KLF4 (Figure 4C, red), OCT4 (Figure 4D, green) and c-MYC (Figure 4E, green) were expressed on both the SMA^+^ pericyte (Figures 4A–C, green) and ERG^+^ endothelial (Figures 4D,E, red) layers of the microvessels in all MG lesions examined. Negative isotype controls, demonstrated minimal staining as expected (Figure 4F).

## Discussion

MG has been generally assumed to be of neuroectodermal origin arising from arachnoid cap cells (2). The first histogenetic concepts favored a dural origin for MG based on its dural attachment. Bright in 1831 noticed the histologic similarities between MG cells and arachnoid villi cells. Cleland and Robin in 1864 first proposed that MG is derived from arachnoid cells, confirmed by Schmidt in 1902 by identifying ultrastructural similarities with respect to cell adhesion mechanisms and components of extracellular matrix (23). More recently Yamashima et al (24) confirm similarities in ultrastructure, cell adhesion mechanisms and extracellular matrix components. While similarities have been demonstrated between arachnoid cap and MG cells, a direct lineage has yet to be determined.

Tumor stem cells are the proposed origin cell of benign and malignant tumors (9). The tumor stem cell concept proposes that a population of stem cells; exist within all tissues and these cells are capable of self-renewal escape from control and form tumors (25). This concept has gained ground with CSCs identified in many malignant tumors including GB (21) and leukemia (4). Interest has developed in identifying the presence of stem cells within benign tumors such as MG with recent reports on the existence of a stem cell population within MG (1, 7, 8). The concept of a hierarchy of stem cell markers recently described in a review article on GB (3) proposes that certain stem cell markers, such as OCT4, are expressed by the most upstream and therefore more primitive stem cells, whereas markers such as vimentin are expressed by more downstream and less primitive, progenitor cells. These authors have described expression of downstream stem cell markers such as vimentin, nestin and CD133 within MG (1, 7, 8). Upstream markers including NANOG, c-MYC, KLF4 and SOX2 have had limited investigation (10, 11, 14). We have demonstrated the expression of these upstream markers including OCT4, a very primitive stem cell marker, within the MG. This supports the notion of the existence of a stem cell population within MG including the most primitive subpopulation.

This report demonstrates the localization of five key ESC markers within the endothelial and pericyte layers of the microvessels within WHO grade I MG suggesting the presence of a primitive population. It is intriguing that tumor stem cells, the proposed origin of many tumors (9) including MG, have been reported to express the markers presented in this report. We postulate that the cells of both the endothelial and the pericyte layers of the microvessels in WHO grade I MG may represent the tumor stem cells and hence the putative origin of MG.

Previous reports have demonstrated a neuro-ectodermal origin for avian brain pericytes (26), with more recent evidence supporting the endothelial cells being the progenitors of the vascular smooth muscle cells (27). It is therefore exciting to speculate that the microvessels within WHO grade I MG, potentially derived from neural crest may be the true origin of MG, which then differentiate into the pericyte and MG tumor cells. However, this is beyond the scope of this study. Another plausible explanation is that these microvessels represent a state of vascular mimicry (28) within the niche of MG tumor stem cells.

## Conclusion

This report presents the novel finding of the expression of ESC-associated markers on the microvessels of WHO grade I MG, inferring that a primitive population within the microvessels are the origin of these tumors. However, larger studies and functional experiments are required to conclusively determine this paradigm.

## Author contributions

TI and ST formulated the study hypothesis. TI, AW, and ST designed the study. TI, AW, HB, GS and ST interpreted the DAB IHC data. TI, AW, and ST interpreted the IF IHC data. TI and ST interpreted the NanoString analysis data. RM performed the statistical analysis. GS drafted the manuscript. All authors commented on and approved the manuscript.

### Conflict of interest statement

TI and ST are inventors of the PCT patents Cancer Diagnosis and Therapy (No. PCT/NZ2015/050108) and Cancer Therapeutic (PCT/NZ2018/050006), and provisional patent application Novel Pharmaceutical Compositions for Cancer Therapy (US/62/711709). The remaining authors declare that the research was conducted in the absence of any commercial or financial relationships that could be construed as a potential conflict of interest.
